# Comparative molecular and conventional cytogenetic analyses of three species of *Rhinella* (Anura; Bufonidae)

**DOI:** 10.1371/journal.pone.0308785

**Published:** 2024-08-15

**Authors:** David Santos da Silva, Rodrigo Petry Corrêa de Sousa, Marcelo Vallinoto, Marlon Ramires da Costa Lima, Renato Araújo da Costa, Ivanete de Oliveira Furo, Anderson José Baia Gomes, Edivaldo Herculano Corrêa de Oliveira

**Affiliations:** 1 Programa de Pós-Graduação em Genética e Biologia Molecular, Instituto de Ciências Biológicas, Universidade Federal do Pará, Belém, Pará, Brazil; 2 Laboratório de Evolução, Instituto de Estudos Costeiros, Universidade Federal do Pará, Bragança, Pará, Brazil; 3 Laboratório de Biologia Molecular, Evolução e Microbiologia, Instituto Federal do Pará, Abaetetuba, Pará, Brazil; 4 Laboratório de Reprodução Animal, Universidade Federal Rural da Amazônia, Parauapebas, Pará, Brazil; 5 Faculdade de Ciências Naturais, Instituto de Ciências Exatas Naturais e Exatas, Universidade Federal do Pará, Belém, Pará, Brazil; 6 Laboratório de Citogenômica e Mutagênese Ambiental, Seção de Meio Ambiente, Instituto Evandro Chagas, Ananindeua, Pará, Brazil; University of Iceland, ICELAND

## Abstract

The genus *Rhinella* corresponds to a group of anurans characterized by numerous taxonomic and systemic challenges, leading to their organization into species complexes. Cytogenetic data for this genus thus far are limited to the diploid number and chromosome morphology, which remain highly conserved among the species. In this study, we analyse the karyotypes of three species of the genus *Rhinella* (*Rhinella granulosa*, *Rhinella margaritifera*, and *Rhinella marina*) using both classical (conventional staining and C-banding) and molecular (FISH-fluorescence *in situ* hybridization with 18S rDNA, telomeric sequences, and microsatellite probes) cytogenetic approaches. The aim of this study is to provide data that can reveal variations in the distribution of repetitive sequences that can contribute to understanding karyotypic diversification in these species. The results revealed a conserved karyotype across the species, with 2n = 22 and FN = 44, with metacentric and submetacentric chromosomes. C-banding revealed heterochromatic blocks in the pericentromeric region for all species, with a proximal block on the long arms of pairs 3 and 6 in *R*. *marina* and on the short arms of pairs 4 and 6 in *R*. *margaritifera*. Additionally, 18S rDNA probes hybridized to pair 5 in *R*. *granulosa*, to pair 7 in *R*. *marina*, and to pair 10 in *R*. *margaritifera*. Telomeric sequence probes displayed signals exclusively in the distal region of the chromosomes, while microsatellite DNA probes showed species-specific patterns. These findings indicate that despite a conserved karyotypical macrostructure, chromosomal differences exist among the species due to the accumulation of repetitive sequences. This variation may be attributed to chromosome rearrangements or differential accumulation of these sequences, highlighting the dynamic role of repetitive sequences in the chromosomal evolution of *Rhinella* species. Ultimately, this study emphasizes the importance of the role of repetitive DNAs in chromosomal rearrangements to elucidate the evolutionary mechanisms leading to independent diversification in the distinct phylogenetic groups of *Rhinella*.

## Introduction

The Bufonidae family is a monophyletic group of anurans, comprising 54 genera and 647 species, with native representatives distributed across almost every continent except in some countries, such as Australia, New Guinea, and Madagascar, where *Rhinella marina* was introduced [1, 2].

In Brazil, Bufonidae is represented by eight genera, with *Rhinella* being the most representative, with 43 species distributed throughout the national territory [3]. Owing to poorly clarified systematics and insufficient morphological, ecological, and molecular data, the genus *Rhinella* has undergone numerous taxonomic changes at both the interspecific and intraspecific levels. Discussions regarding the true taxonomic status of certain species have ensued, leading many to be classified within species complexes, thereby underscoring the taxonomic challenges associated with this genus [4–8].

The genus *Rhinella* comprises three major species complexes: *Rhinella margaritifera*, *Rhinella granulosa*, and *R*. *marina*. Despite extensive efforts and a wealth of studies across diverse areas, uncertainties and inconsistencies persist within these groups, with new species being continually described [6–11].

Few studies have tackled the cytogenetics of the genus *Rhinella*, primarily involving conventional staining, banding, and few molecular analyses. These investigations revealed a remarkable conservatism in diploid numbers and chromosome morphology across the most distinct complexes, such as *Rhinella marina*, *Rhinella margaritifera*, *Rhinella granulosa*, and *Rhinella crucifer* [8, 12, 13]. Notably, no divergences have been detected even in the patterns of C-banding, NOR (nucleolar organizer region), or fluorescence *in situ* hybridization (FISH) with probes from 18S rDNA [12, 13].

An alternative approach to understanding the evolutionary mechanisms associated with karyotypic diversification is the analysis of different repetitive sequences, such as microsatellites, telomeric sequences, transposition elements, and more. These sequences play a crucial role in genome organization and plasticity and serve as excellent chromosomal markers in comparative cytogenomics [14–18].

Repetitive sequences are abundant in the genomes, and each species possesses a specific library of repetitive element families, categorized as satellite DNAs, minisatellites, microsatellites, transposable elements, and multigenic families of ribosomal RNA genes [19]. Notably, microsatellite sequences have been highlighted for their significance. The physical mapping of the accumulation of microsatellite sequences has proven valuable in identifying sexual systems in amphibians, providing new insights into the mechanisms of genomic and karyotypic evolution [12, 16, 20].

In this study, we aimed to analyse the organization of repetitive DNA sequences in species representing the *R*. *granulosa*, *R*. *margaritifera*, and *R*. *marina* complexes using banding techniques and fluorescence *in situ* hybridization experiments, which contributed to a better understanding of karyotypic diversification and cytotaxonomy within the analysed species.

## Materials and methods

### Specimen collection, preparations, and chromosome banding

**Fig 1:**
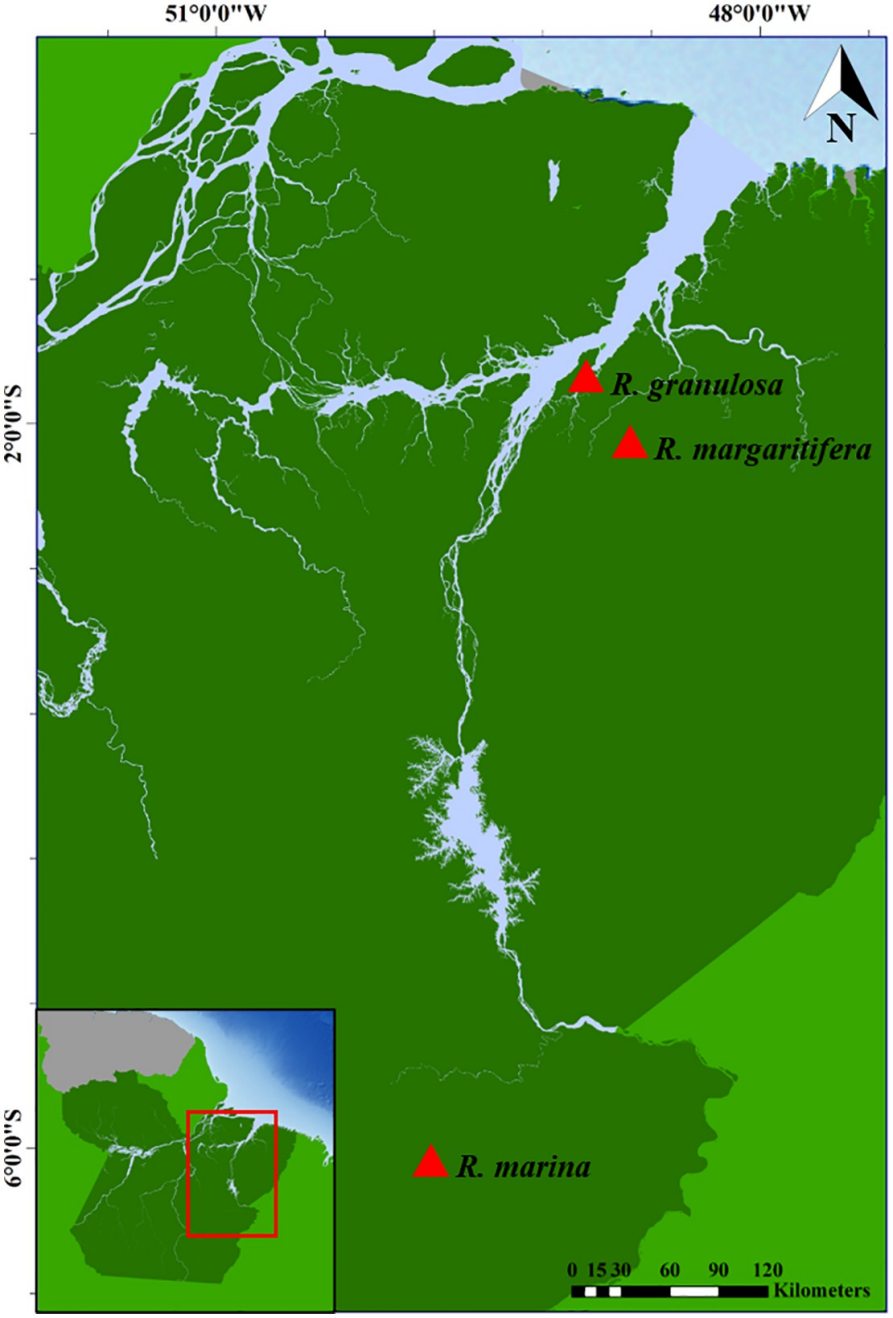
Specimen collection sites of *R*. *granulosa*, *R*. *margaritifera*, and *R*. *marina* in the Amazon rainforest, Pará, Brazil. The red triangles highlight the collection sites of the species analysed in this study. Map produced in QGIS software, version 3.36 (https://qgis.org/pt_BR/site/), input data are public domain obtained from the Instituto Brasileiro de Geografia e Estatística (https://www.ibge.gov.br/geociencias/downloads-geociencias.html).

For this study, three species of *Rhinella* were collected from areas within the Amazon rainforest in northern Brazil (permission SISBIO licence n° 78948, CEUA authorization N° 3539290620): *R*. *granulosa* (4 males and 4 females) (1°44’08.3"S 48°57’31.5"W), *R*. *margaritifera* (1 female) (2°05’49.0”S; 48°43’00.2”W), and *R*. *marina* (1 male and 3 females) (6°03’50.1"S 49°48’55.1"W) (Fig 1). Specimens were properly identified using morphological criteria described by Kwet et al. [21], Narvaes and Rodrigues [22], and Lavilla et al. [23]. Subsequently, the samples were deposited in the zoological collection of the Instituto Federal do Pará (Abaetetuba, PA).

The specimens were euthanized with cutaneous applications of 2% lidocaine with the consent of the Ethical Committee in Animal Use (permission number 3539290620). Chromosome preparations were obtained from the intestinal epithelium and bone marrow following the protocols proposed by Ford and Hamerton [24] and Schmid [25], respectively. For male specimens, chromosomal preparations of gonads were also obtained from the testes according to Ford and Hamerton [24]. For conventional cytogenetic analysis, chromosomes were stained with 5% Giemsa solution at pH 6.8 (0.5 ml of Giemsa supplemented with 10 ml of Phosphate buffer), while C-banding followed Sumner [26], with modifications in relation to the exposure time in barium hydroxide, where exposure varied between 1.5 minutes and 2 minutes, and the final staining where we used Wright stain.

### Fluorescence *in situ* hybridization (FISH)

The 18S rDNA and telomeric sequences were amplified from the DNA of *R*. *marina* using the primers 18Sf (5’-CCGAGGACCTCACTAAACCA-3’) and 18Sr (5’-CCGCTTTGGTGACTCTTGAT-3’) [27], resulting in a 1400-bp PCR product. Telomeric (TTAGGG)*n* sequences were generated via PCR using the (TTAGGG)_5_ and (CCCTAA)_5_ primers without a DNA template, as described by Ijdo et al. [28]. Because it is a highly conserved sequence among vertebrates, we opted not to sequence the 18S rDNA PCR product. The 18S rDNA and telomeric sequence probes were labelled by nick translation with digoxigenin-dUTP (Roche, Mannheim, Germany) following the manufacturer’s recommendations. The signals from the probes were detected using an antidigoxin antibody with fluorescein (green) or rhodamine (red). FISH experiments with the aforementioned repetitive sequences were conducted following the protocol described by Yano et al. [29].

Concerning the microsatellite sequences, 11 di/trinucleotide repeats were used as probes: (CA)_15_, (GA)_15_, (TA)_15_, (GC)_15_, (CAA)_10_, (CAC)_10_, (CAG)_10_, (CAT)_10_, (CGG)_10_, (GAA)_10_, and (GAG)_10_, following the procedures adopted by Kubat et al. [30], with modifications as described by Cioffi et al. [31]. All probes used were commercially obtained and labelled directly with Cy3 in the 5’ terminal region during synthesis (Sigma, St. Louis, MO, USA).

### Microscopic analysis and image processing

A total of 20 metaphases per experiment were analysed to determine the diploid number, chromosome morphology, distribution of heterochromatic blocks, and patterns of distribution of the repetitive sequences. The metaphases with optimal dispersal were captured under a Leica 1000 DM microscope using a 100x objective. Karyotypes were organized using GenASIs software, version 7.2.6.19509 (Applied Spectral Imaging, California, USA). The results of the FISH experiments were registered using a Zeiss Axio ImagerZ.2 epifluorescence microscope, and images were captured and edited with AxioVision 4.8 software (Zeiss, Jena, Germany).

Fundamental numbers (FNs) were calculated based on the total number of chromosome arms, considering metacentric (m), submetacentric (sm), and subtelocentric (st) as biarmed chromosomes and telocentric (t) as uniarmed chromosomes, according to the classification proposed by Green and Sessions [32].

## Results

### Karyotyping and banding

**Fig 2:**
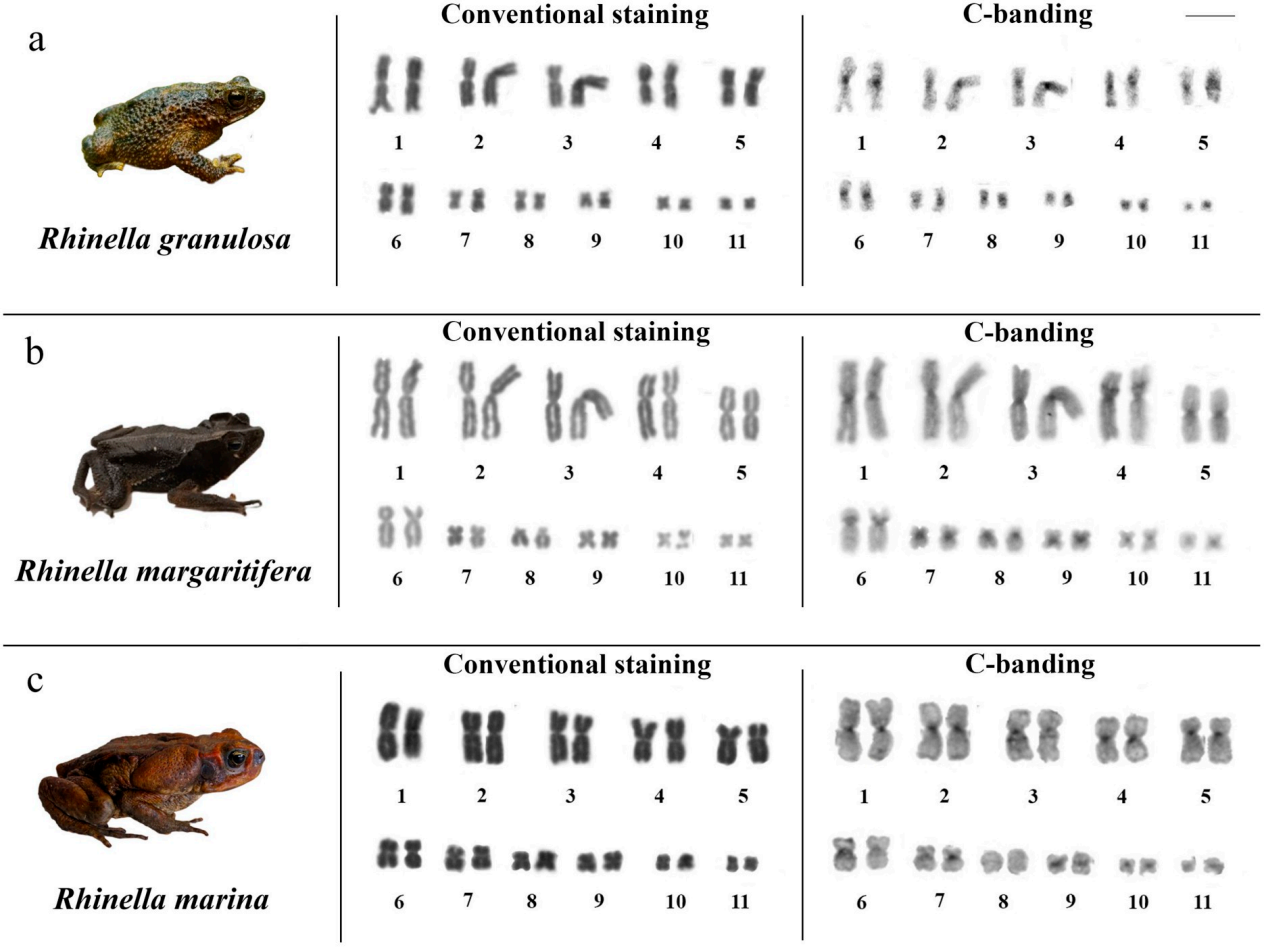
Karyotype with conventional staining and C-banding of the species a) *R*. *granulosa*, b) *R*. *margaritifera*, and c) *R*. *marina* (top to bottom). The chromosomes were arranged in decreasing order after Giemsa staining. Scale bar = 10 μm.

All analysed species exhibited a diploid number of 2n = 22 chromosomes, resulting in a fundamental number (FN) of 44 (Fig 2). The karyotype of *R*. *granulosa* consisted of eleven metacentric pairs, while *R*. *margaritifera* showed nine metacentric pairs (1, 2, 3, 4, 5, 7, 9, 10, and 11) and two submetacentric pairs (6 and 8), and *R*. *marina* had ten metacentric pairs (1, 2, 3, 4, 6, 7, 8, 9, 10, and 11), and only one submetacentric pair (5) (S1 Table). Furthermore, no sexual dysmorphism was observed among the karyotypes of the species analysed. Interspecific morphological variations were observed in certain chromosome pairs, notably in pair 10 of *R*. *margaritifera*. C- banding revealed heterochromatic blocks in the centromeric region for all species, with a conspicuous accumulation in the pericentromeric region of the short arms of pairs 4 and 6 in *R*. *margaritifera* and in the long arms of pairs 3 and 6 in *R*. *marina*. (Fig 2).

### FISH experiments

**Fig 3:**
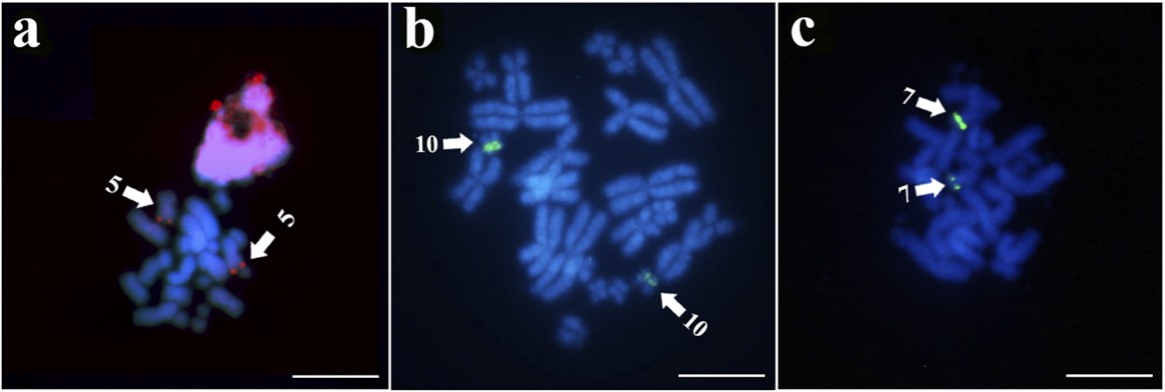
FISH with 18S rDNA probes of the species a) *R*. *granulosa*, b) *R*. *margaritifera*, and c) *R*. *marina*. The arrows indicate the chromosomes that showed signals of hybridization with the 18S rDNA probe. The chromosomes were counterstained with DAPI (blue). Scale bar = 10 μm.

**Fig 4:**
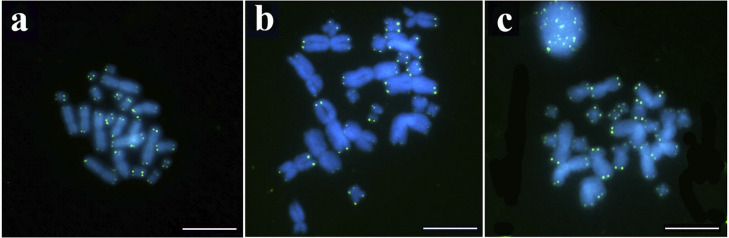
FISH with telomeric sequences of (TTAGGG)*n* probes of the species a) *R*. *granulosa*, b) *R*. *margaritifera*, and c) *R*. *marina*. The chromosomes were counterstained with DAPI (blue). Scale bar = 10 μm.

The 18S rDNA probe showed signals in the distal regions of the long arm of pair 5 of *R*. *granulosa*, in the interstitial region of the short arm of pair 7 in *R*. *marina*, and in the subdistal region of the short arm of pair 10 in *R*. *margaritifera* (Fig 3). Hybridization with telomeric sequence probes produced signals exclusively in the distal region of the chromosomes (Fig 4).

**Fig 5:**
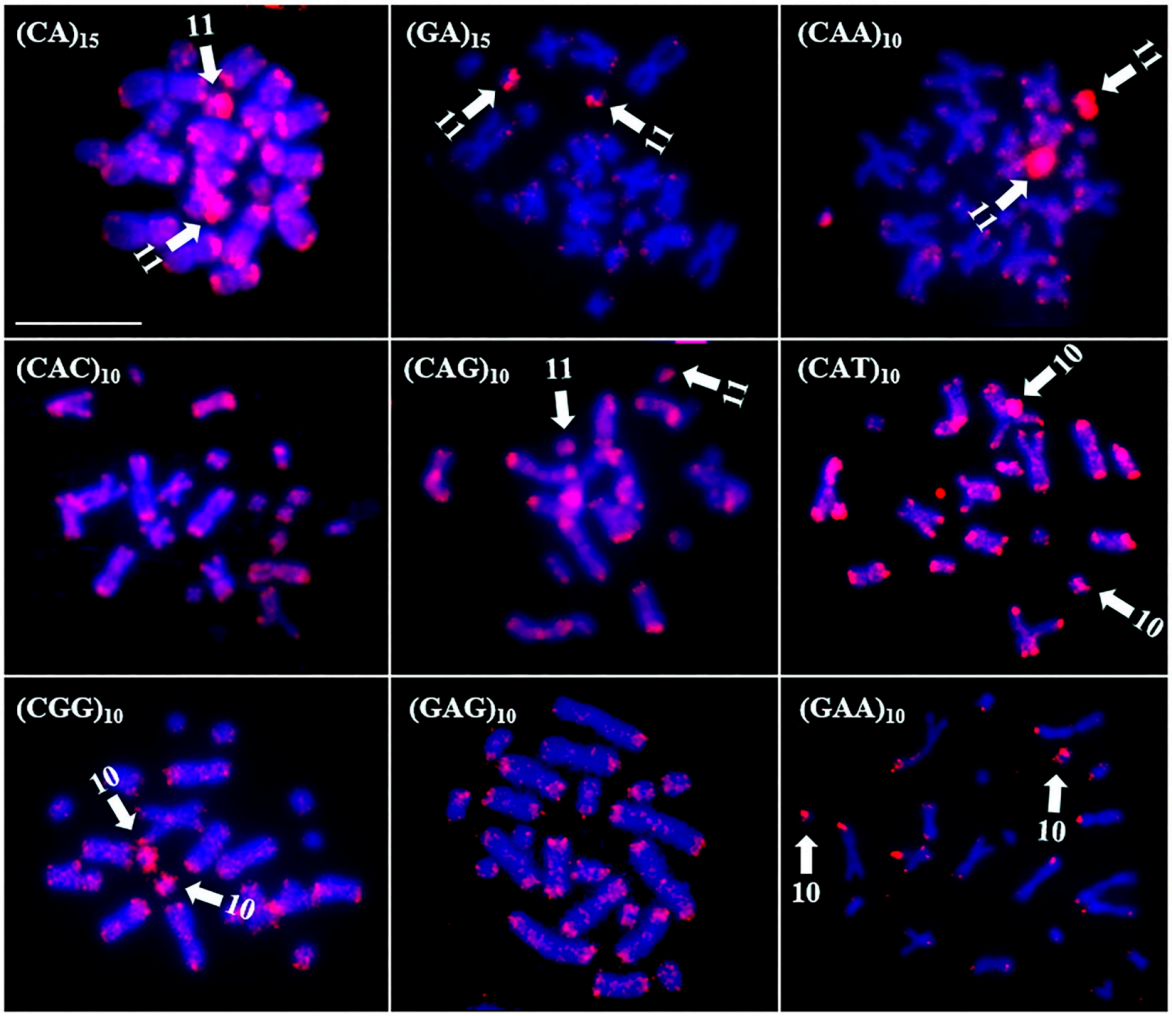
Distribution of microsatellites in the genome of *R*. *granulosa*. The microsatellite probes used are indicated at the top left. The arrows indicate the chromosomes that showed specific signs of hybridization with the microsatellite probe used. The chromosomes were counterstained with DAPI (blue). Scale bar = 10 μm.

The microsatellite probes produced two different patterns of hybridization in *Rhinella* species: scattered signals or signals in specific regions of the chromosome. In *R*. *granulosa*, nine probes produced signals. In general, all the probes hybridized to the distal portion of all the chromosomes, with some probes also showing chromosome-specific signals (Fig 5). The probes (CAT)_10_, (CGG)_10_, and (GAA)_10_ accumulated in pair 10, and probes (CA)_15_, (GA)_15_, (CAA)_10_, and (CAG)_10_ accumulated in pair 11 (Fig 5).

**Fig 6:**
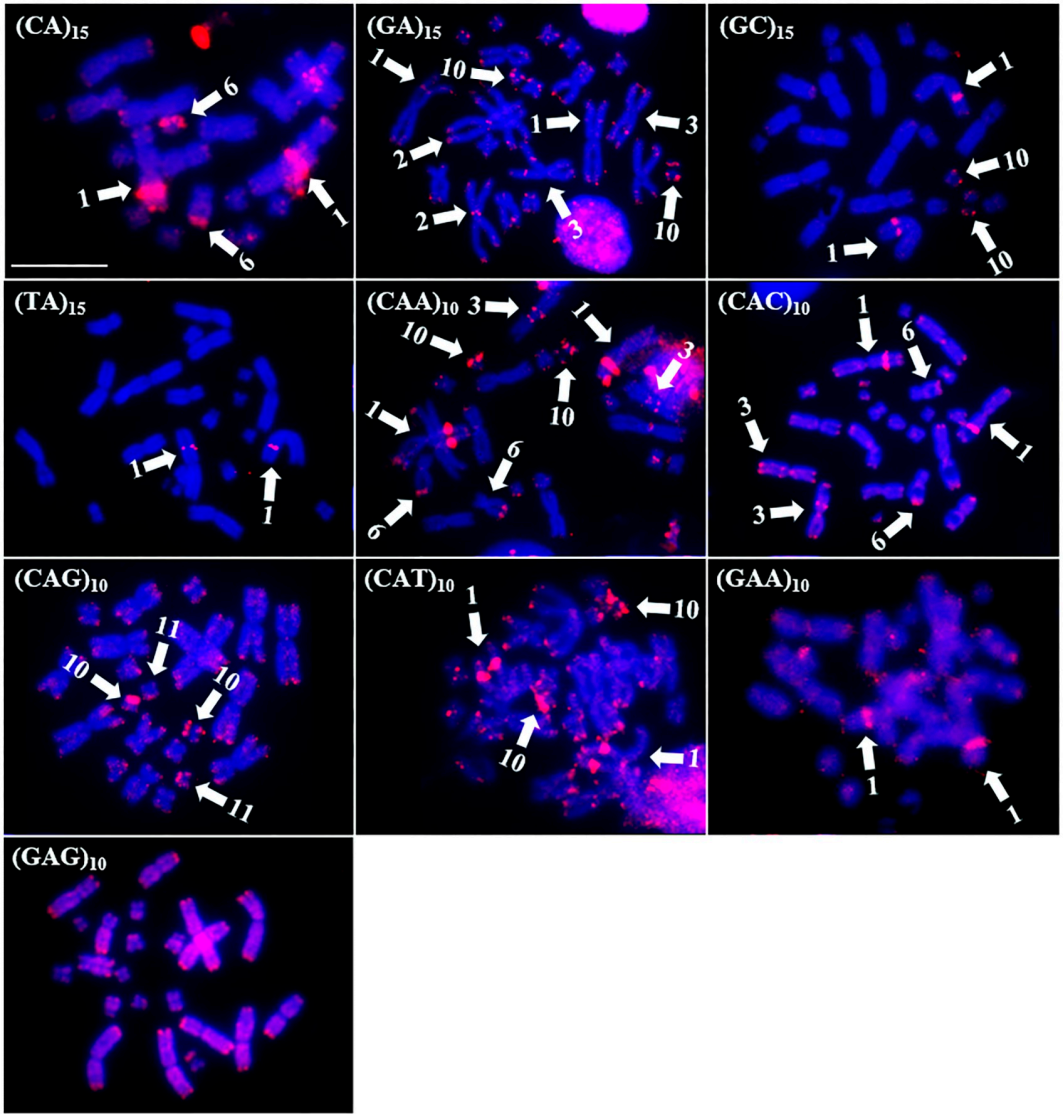
Distribution of microsatellites in the genome of *R*. *margaritifera*. The microsatellite probes used are indicated at the top left. The arrows indicate the chromosomes that showed specific signs of hybridization with the microsatellite probe used. The chromosomes were counterstained with DAPI (blue). Scale bar = 10 μm.

On the other hand, in *R*. *margaritifera*, ten probes produced signals. The (CA)_15_, (GA)_15_, (CAA)_10_, (CAC)_10_, (CAG)_10_, (CAT)_10_, (GAA)_10_, and (GAG)_10_ probes hybridized mainly to the distal portion of the chromosomes (Fig 6). In addition, hybridization-specific signals from the (CA)_15_, (GA)_15_, (GC)_15_, (TA)_15_, (CAA)_10_, (CAC)_10_, (CAT)_10_, and (GAA)_10_ sequences were observed in the interstitial region of the long arm of pair 1 (Fig 6). Some hybridization signals in the centromeric region were observed with the (GA)_15_ probe in pair 2, while the (GA)_15_ and (CAA)_10_ probes revealed interstitial hybridization signals in the short arm of pair 3. The (CA)_15_, (CAA)_10_, and (CAC)_10_ probes showed signal accumulation in the distal portion of the long arm of pair 6 (Fig 6).

Other intense hybridization signals were observed on chromosome 10 with the (GA)_15_, (GC)_15_, (CAA)_10_, (CAG)_10_, and (CAT)_10_ probes, and on pair 11, the (CAG)_10_ probe also generated intense hybridization signals. Only the (GAG)_10_ probe did not show a specific hybridization pattern, hybridizing solely in the distal region and displaying dispersed signals in the euchromatic region (Fig 6).

**Fig 7:**
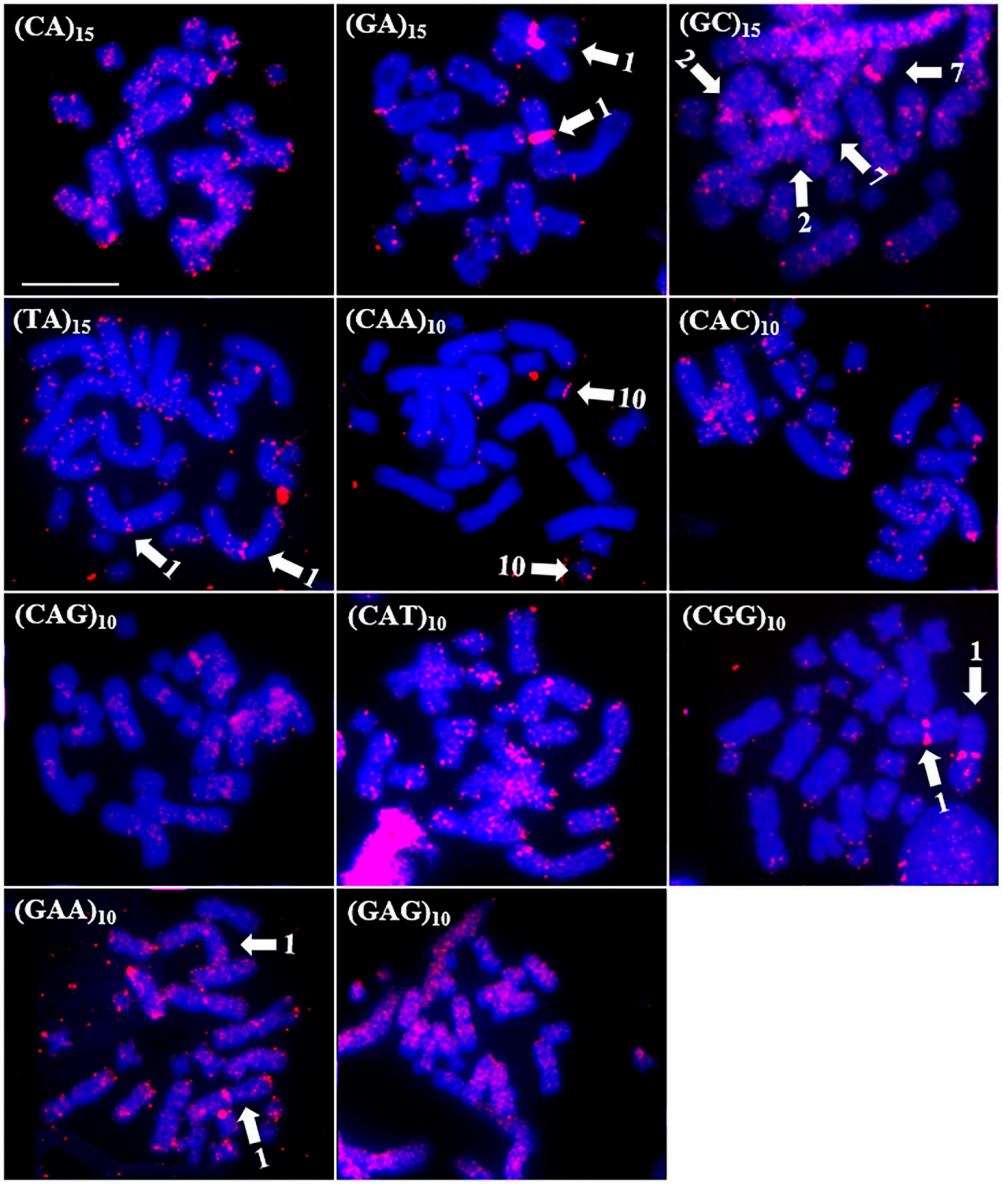
Distribution of microsatellites in the genome of *R*. *marina*. The microsatellite probes used are indicated at the top left. The arrows indicate the chromosomes that showed specific signs of hybridization with the microsatellite probe used. The chromosomes were counterstained with DAPI (blue). Scale bar = 10 μm.

In *R*. *marina*, all the microsatellite probes (a total of eleven) hybridized to the chromosomes of the species. The probes (CA)_15_, (CAC)_10_, and (GAA)_10_ produced signals in the distal regions; (CAG)_10_ produced signals in the proximal regions; and the (GAG)_10_ and (CAT)_10_ probes produced scattered signals (Fig 7). The (CGG)_10_, (GA)_15_, (GAA)_10_, and (TA)_15_ probes exhibited specific hybridization signals in the proximal region of pair 1 and in some chromosomes in the distal region. Moreover, the (CAA)_10_ probe hybridized in the distal region of the short arm of pair 10, and probe (GC)_15_ revealed signals in the proximal regions of pairs 2 and 7 (Fig 7).

## Discussion

The genus *Rhinella* comprises a great diversity organized into species complexes due to their high morphological similarity and complex phylogenetic relationships [4, 11, 33–35]. Although cytogenetic data obtained via classical approaches have been previously described for the analysed species, this study represents the first application of probes targeting different repetitive sequences to understand the genomic organization of *Rhinella* species.

The conserved karyotypic status observed among the species/complexes within the genus *Rhinella* has been a significant puzzle. Conventional chromosome analyses of the species in this study reaffirmed the conservation of the macrostructure of the *Rhinella* species karyotype. Bruschi et al. [13] reported a common karyotype with 2n = 22 and FN = 44 across all the species, albeit with minor variations in chromosome morphology. This observation led us to consider that the events resulting in morphological chromosomal changes occurred independently in each lineage of the species group, potentially involving the participation of repetitive sequences.

It is worth noting that the diploid number of 2n = 22 is also a recurrent finding in anurans in general, possibly corresponding to a plesiomorphic characteristic of the order [36]. This chromosomal conservatism has evolutionary implications, as chromosomal characteristics can act as important pre- or postzygotic barriers to reproduction among distinct species [13, 37]. In this case, the chromosomal similarity between species would result in a relaxed isolation mechanism for speciation, contributing to the observed high frequency of hybridization events between species of the genus [11, 13, 33, 34].

Moreover, other aspects of chromosome structure also exhibit uniformity in *Rhinella*. For example, although the PCR product has not been sequenced, our results with 18S rDNA corroborated previous data obtained from silver staining, confirming the presence of a nucleolar organizer region in pair 5 of *R*. *granulosa*, pair 7 of *R*. *margaritifera*, and pair 10 of *R*. *marina* [13]. This allows inference of interspecific chromosomal homologies within species of this complex. This interspecific concordance is also observed in all species of the *R*. *granulosa* complex (distal portion of the long arm of pair 5), *R*. *marina* complex (interstitial portion of the short arm of pair 7), and *R*. *margaritifera* complex (subdistal portion of the short arm of pair 7 or 10), and suggests that the 18S rDNA probe obtained by PCR corresponded to the specific sequence of the genes [12, 13, 38–40].

The dynamics of the location of 18S rDNA cluster probes across different species complexes may result from intra- and interchromosomal rearrangements, including inversions, fusions, and translocations, as well as transposition element-mediated transposition events or error reinsertion during amplification events [17, 38, 41, 42]. Therefore, this specificity within each group may represent a putative synapomorphy for each of them, except for the *R*. *margaritifera* group. In this group, NOR and 18S rDNA are found either in pair 7 or in pair 10, suggesting a reversion of the character or retention of the ancestral polymorphism, according to Bruschi et al. [13].

On the other hand, an alternative hypothesis that can be raised to justify these divergences in relation to the position of NORs and 18S rDNA is the variation in the copy number of tandem repeats/multigene families [43, 44]. Such variation can explain, for example, the differences observed both at the intraspecific level in *R*. *margaritifera* and at the interspecific level in *Rhinella* species, in which these markers are distributed at different positions.

Fornani et al. [44] reported that the differences in the number of copies of repetitive sequences of U1 and U2 snDNA were the result of the loss or reduction in the number of copies of these sequences in the different *Xenopus* (pipid frogs) species analysed. In the case of *Rhinella* species, the expansion of tandem repeats may have been an important driver of evolution following rearrangements such as translocation, inversion, deletion, and degeneration, which could explain the different locations of the repetitive sequences in the different *Rhinella* species.

Another informative chromosome marker in studies of karyotypic diversification in anurans is the distribution of heterochromatic blocks. Heterochromatin can serve as a hotspot for chromosomal rearrangements, and therefore, a detailed analysis of its composition and distribution enhances our understanding of karyotype evolution dynamics [12, 18, 20, 45]. Although C-banding analyses in species of the genus *Rhinella* are relatively limited, studies up to the level of the family Bufonidae suggest a highly conserved banding pattern, with these blocks primarily restricted to centromeres and pericentromeric regions [12, 46].

While accumulations of heterochromatin in pericentromeric regions in pairs 3 and 6 in *R*. *marina* and pairs 4 and 6 in *R*. *margaritifera* may suggest rearrangements, studies with species of the genus *Rhinella* and other Bufonids have considered such findings as potential population markers within Bufonidae [47, 48]. Notably, extensive heterochromatic blocks observed in the chromosome pairs of the species *R*. *marina* and *R*. *margaritifera* indicate the amplification of repeat units, underscoring the role of repetitive DNAs in *Rhinella* chromosome evolution and, consequently, in karyotypic divergences among species [49].

**Fig 8:**
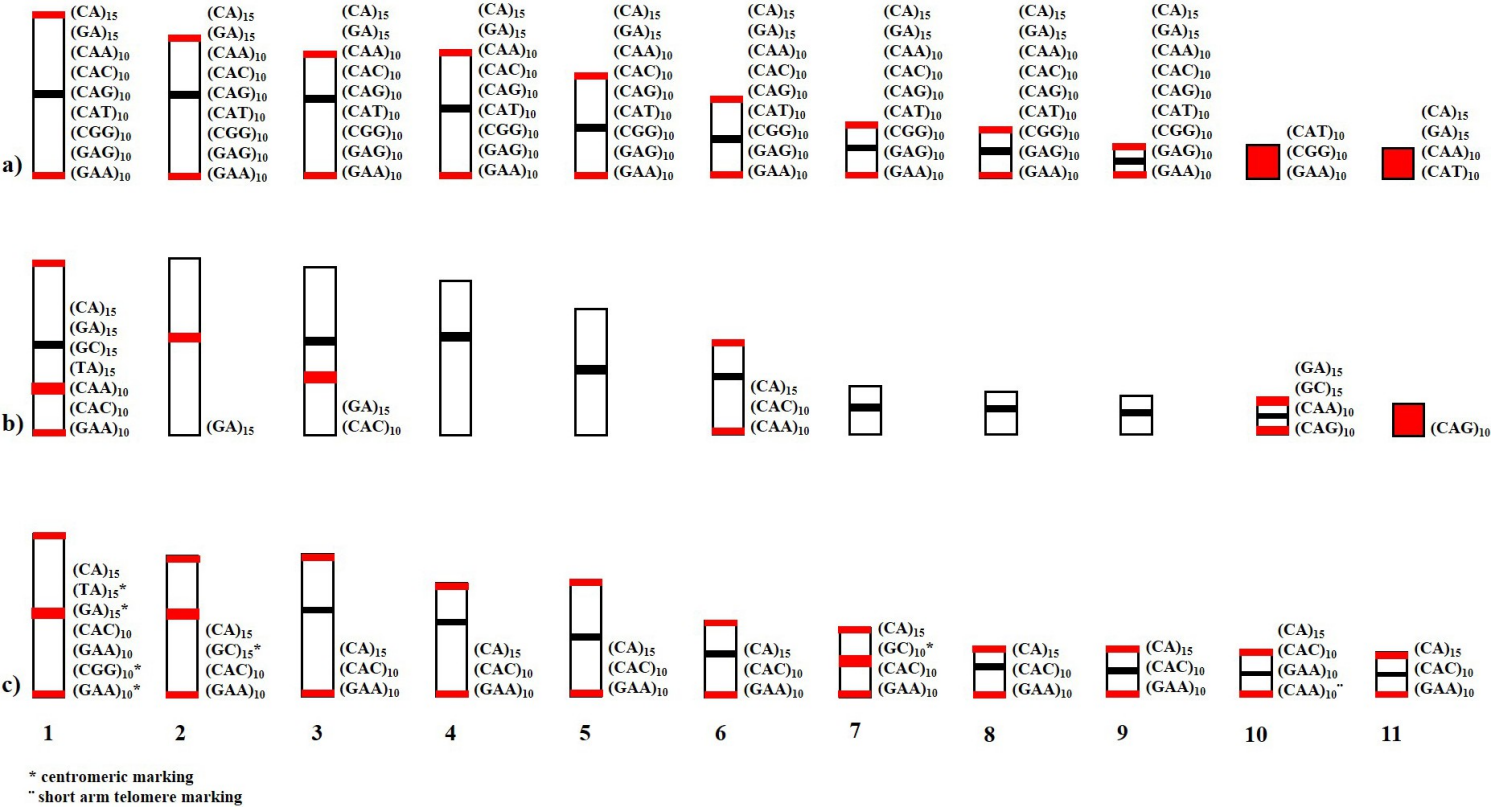
Distribution scheme of the main microsatellites on the autosomal chromosomes of a) *R*. *granulosa*, b) *R*. *margaritifera*, and c) *R*. *marina*. The red markers indicate the distribution of the microsatellites specific to each chromosome. The black markers indicate the centromeric position.

In recent years, several studies have reported that certain species exhibit specific markers that may play regulatory roles in gene activities and genomic functions [50, 51]. In the case of species of the genus *Rhinella*, despite is phylogenetically related, and diverse patterns in the location of microsatellite repeats have been identified. These differences suggest potential variations in evolutionary events of genomic organization, with some microsatellite accumulations being species-specific (Fig 8) [52, 53].

Studies have reported that microsatellites are not randomly distributed in eukaryotic genomes and may be in the same chromosomal locations in closely related species [18, 54, 55]. Indeed, the distributions of microsatellites in the species *R*. *marina* and *R*. *granulosa*, which are phylogenetically more closely related, were more similar than those in *R*. *margaritifera*, which occupies a more basal position in the phylogeny of the genus *Rhinella*, displaying more distinct patterns of microsatellite distribution. These results reinforce the hypothesis that microsatellite distribution can provide phylogenetic markers depending on the groups and species studied [11].

The specific accumulation of microsatellites on heteromorphic sex chromosomes is common due to the appearance of nonrecombinant regions. In addition, significant accumulations of microsatellite sequences can also occur in euchromatic regions and not necessarily in sex-linked regions/chromosomes, and in turn, such cytogenetic markers could play a role in modulating genomic function [17, 51, 56]. Given this context, two interesting aspects should be raised: 1- the accumulation of microsatellite sequences in pair 1 of *R*. *marina* and *R*. *margaritifera*; 2- the dimorphism of pair 10 of *R*. *margaritifera*, as well as the accumulation of microsatellites in this same pair in both *R*. *granulosa* and *R*. *margaritifera*.

Specific accumulations in pair 1 have been reported in some species of Bufonidae [57, 58]. Interestingly, molecular studies have revealed that in four species of the genus *Bufo*, genes associated with sex definition are present on chromosome pair 1 [57, 59]. Furthermore, a recent study based on genomic data revealed numerous sex-linked markers, located throughout chromosome 1, with some markers also linked to chromosome 7. Overall, this provides strong support for a genetic sex determination system on chromosome 1 [58]. However, no information on the accumulation of repetitive sequences or sex-defining genes in pair 10 of Bufonidae has been described. Unfortunately, the lack of genomic data available for the species analysed limits us from suggesting that such markers may have some functionality in identifying sex chromosomes in *Rhinella* species and that more sophisticated genomic analyses, such as comparative genomic hybridization or next-generation sequencing, should be carried out to address these uncertainties.

Interestingly, in the karyotypes of the three species, the trinucleotide probes (CAC)_10_, (CAT)_10_, and (GAG)_10_ showed a dispersed distribution pattern throughout the chromosomes. Such a distribution of microsatellite sequences throughout genomes has been associated with the activity of transposable elements, which may contain microsatellite repeats in their sequences, thus contributing to the dispersion of units during transposition events and influencing the karyotypic diversification processes of the species [60, 61].

In summary, our data suggest that repetitive DNAs play a dynamic role in chromosomal changes in *Rhinella*, influencing the chromosomal microstructure and contributing to our understanding of the evolutionary mechanisms that led to karyotypical diversification in distinct phylogenetic groups within this genus.

## Conclusions

While at the macrochromosomal level, species within the genus *Rhinella* exhibit apparent conservatism, cytogenetic mapping of different repetitive DNA sequences has provided significant chromosomal markers, revealing species-specific differences. Furthermore, chromosomal mapping of repetitive DNAs in these species has expanded our ability to recognize karyological features that cannot be discerned using classical cytogenetic methods. From an evolutionary perspective, we can speculate that these chromosomal features may have been involved in the genomic diversification of the *Rhinella* group, reinforcing the importance of exploring different aspects of repetitive sequences in analyses of cytogenetic composition and evolution.

## Supporting information

S1 Table Morphometric data of mitotic chromosomes of *Rhinella* species analysed. Classification by Green and Sessions [32].
(DOCX)
