# Supplementary material for: Comparative molecular and conventional cytogenetic analyses of three species of Rhinella (Anura; Bufonidae)
Source: PLoS One. 2024 Aug 15;19(8):e0308785. doi: 10.1371/journal.pone.0308785 (PMC11326569; doi:10.1371/journal.pone.0308785)
Supplement: S1 Table — Classification by Green and Sessions [32]. (DOCX) [file pone.0308785.s001.docx]

**S1 Table.** **Morphometric data of mitotic chromosomes of *Rhinella* species analysed.** Classification by Green and Sessions [32].

| **Chromosome Pair** | | **1** | **2** | **3** | **4** | **5** | **6** | **7** | **8** | **9** | **10** | **11** |
| --- | --- | --- | --- | --- | --- | --- | --- | --- | --- | --- | --- | --- |
| **RGR** | CR | 1.20 | 1.29 | 1.22 | 1.21 | 1.12 | 1.04 | 1.15 | 1.23 | 1.24 | 1.13 | 1.08 |
|  | CI | 0.46 | 0.43 | 0.46 | 0.45 | 0.47 | 0.49 | 0.46 | 0.45 | 0.45 | 0.47 | 0.48 |
|  | Morphology | m | m | m | m | m | m | m | m | m | m | m |
| **RMG** | CR | 1.21 | 1.23 | 1.29 | 1.53 | 1.15 | 1.74 | 1.19 | 2.26 | 1.02 | 1.03 | 1.08 |
|  | CI | 0.45 | 0.45 | 0.44 | 0.39 | 0.46 | 0.36 | 0.45 | 0.31 | 0.49 | 0.49 | 0.48 |
|  | Morphology | m | m | m | m | m | sm | m | sm | m | m | m |
| **RMR** | CR | 1.1 | 1.43 | 1.48 | 1.15 | 1.81 | 1.13 | 1.44 | 1.11 | 1.03 | 1.32 | 1.04 |
|  | CI | 0.48 | 0.41 | 0.4 | 0.46 | 0.37 | 0.47 | 0.41 | 0.47 | 0.49 | 0.44 | 0.49 |
|  | Morphology | m | m | m | m | sm | m | m | m | m | m | m |

RGR- *Rhinella granulosa*; RMG- *Rhinella margaritifera*; RMR- *Rhinella marina*; CR- Centromeric ratio; CI- Centromeric index, m- Metacentric (CR= 1-1.67; CI= 0.5-0.375); sm- Submetacentric (CR= 1.68-3; CI= 0.374-0.25).
